# Neutrophil-related gene expression profile is associated with future paediatric bronchiectasis exacerbations

**DOI:** 10.1007/s00109-026-02662-0

**Published:** 2026-04-01

**Authors:** Hannah E. O’Farrell, Vikas Goyal, Anne B. Chang, Keith Grimwood, Michael Cheng, Stephanie T. Yerkovich, Katherine J. Baines

**Affiliations:** 1 NHMRC Centre for Research Excellence in Paediatric Bronchiectasis (AusBREATHE), Child and Maternal Health Division, Menzies School of Health Research, Charles Darwin University, Darwin, NT 0810 Australia; 2 Australian Centre for Health Services Innovation and School of Medicine, Queensland University of Technology, Brisbane, QLD 4000 Australia; 3 Department of Respiratory and Sleep Medicine, Queensland Children’s Hospital, Brisbane, QLD 4101 Australia; 4 Department of Paediatrics, Gold Coast Health, Gold Coast, QLD 4215 Australia; 5 School of Medicine and Dentistry, Griffith University, Gold Coast, QLD 4222 Australia; 6 School of Biomedical Sciences and Pharmacy, The University of Newcastle, Newcastle, NSW Australia

**Keywords:** Bronchiectasis, Paediatrics, Gene expression, Microarray

## Abstract

**Abstract:**

Acute respiratory exacerbations in bronchiectasis are important as they impair quality of life and are associated with accelerated lung function decline. Yet, no validated methods exist to identify children at increased risk of exacerbations. We therefore determined if peripheral blood gene expression (GE) signatures can identify those at risk of an impending exacerbation. Thirty-one children with bronchiectasis had RNA extracted from peripheral blood collected whilst they were clinically stable, with 22 having an exacerbation during the next 3 months. Microarray assays using the HumanHT-12 v4.0 Expression BeadChip identified differentially expressed genes (*p* value ≤ 0.05, fold change > 1.5). The top targets were verified using real-time quantitative polymerase chain reaction (rt-qPCR) assays, and receiver operating characteristics and area under the curve (AUC) were assessed. Functional analysis of these genes was performed using Ingenuity Pathway Analysis. Overall, 647 entities were significantly dysregulated (*p* < 0.05) in the exacerbation group (*n* = 22), and pathway analysis identified neutrophil degranulation as the dominant affected pathway, which was also significantly inhibited (*p* < 0.001). Forty entities (32 genes) were associated with a future exacerbation (*p* ≤ 0.05, fold change ≥ 1.5) and six genes (*ANXA3*, *ALAS2*, *DEFA1*, *ALPL*, *SNCA*, *PROK2*) were verified using RT-qPCR (all *p* < 0.04) as the most discriminatory. *DEFA1* and *ANXA3* had the highest AUC (0.92, 95% confidence interval [CI] 0.82–1.00, and 0.87, 95% CI 0.73–1.00, respectively). We identified neutrophil-associated genes from peripheral blood that could be potential biomarkers for children with bronchiectasis at increased risk of exacerbations during the next 3 months. These GE signatures warrant further investigation and validation in larger, independent cohorts.

**Key messages:**

- Exacerbations in paediatric bronchiectasis are important.
- Peripheral blood gene expression may help identify children at risk of exacerbations.
- Six neutrophil-associated genes were associated with a future exacerbation.
- Identifying predictive gene expression signatures warrants further investigation.

**Supplementary Information:**

The online version contains supplementary material available at 10.1007/s00109-026-02662-0.

## Introduction

Bronchiectasis unrelated to cystic fibrosis is characterised clinically by chronic or recurrent wet/productive cough combined with airway infection, neutrophilic inflammation and abnormal bronchial dilatation on a chest computed tomography scan [1, 2]. It is a major cause of respiratory morbidity and mortality globally, ranking third behind asthma and chronic obstructive pulmonary disease (COPD) as the most common cause of chronic airway disorders in adults [3]. In particular, it impacts upon Indigenous populations in high-income countries, such as Australia where Indigenous Australians with bronchiectasis die at a median age of 50 years, which is 22 years earlier than non-Indigenous Australians with the same pulmonary disorder [4]. Furthermore, bronchiectasis results in substantial economic costs to both the health system and patients. The estimated aggregate annual costs of bronchiectasis in US adults in 2021 was USD14.68 billion, whilst in Australian children it was USD17.77 million [5]. In addition, children and their families with bronchiectasis have impaired quality of life (QoL), especially during exacerbations [6]. Despite the burden of illness, only recently has the importance of bronchiectasis been recognised, especially in children and young people (CYP) where it has received relatively little attention [7, 8]. Unsurprisingly, large knowledge gaps remain, especially for acute exacerbations where there are episodes of increased respiratory symptoms [7, 9]. Exacerbations in CYP with bronchiectasis are clinically important as they are associated with increased stress, impaired QoL [1], disease progression with lung function decline (−1.9% forced expiratory volume in 1-s predicted per hospitalised exacerbation) [10] and substantial healthcare costs (e.g. in Australian children in 2016 it was $32.67 thousand dollars [USD24.26], per hospitalisation) [11]. Moreover, parents, patients and clinicians rate exacerbations among the top clinical and research priorities for CYP with bronchiectasis [12]. Yet, little is known about the pathobiology of exacerbations in paediatric bronchiectasis [7, 9] and there are currently no validated methods to identify CYP at imminent risk of future exacerbations.

Bronchiectasis is an inflammatory airway disease with neutrophil predominance, the most common inflammatory endotype [13–15]. Indeed, high levels of neutrophil elastase in sputum in adults predict increased risk of airway infections and future exacerbations [16]. Novel gene expression (GE) signatures are proven biomarkers and provide disease-specific mechanistic insights in various conditions [17]. GE signatures are also proven biomarkers for acute exacerbations in other chronic airway inflammatory diseases (e.g. asthma [18] and COPD [17]) but have not been studied in paediatric bronchiectasis. We therefore analysed stored blood samples from two prior randomised control trials (RCTs), BEST-1 [19] and BEST-2 [20], to evaluate if differentially expressed genes (DEGs) are associated with an exacerbation within the subsequent 3 months of the blood collection. We hypothesised that children at risk of an imminent (within 3 months) exacerbation will have a distinctive neutrophil-based GE profile. Identifying potential predictive GE signatures is clinically important as this could provide early detection of children at risk of exacerbations and instigate changes in their clinical management.

## Methods

### Study design, participants and sample selection

BEST-1 [19] and BEST-2 [20] were two parallel, multicentre, double-blind, placebo-containing RCTs that examined the efficacy of antibiotics to treat non-severe (non-hospitalised) respiratory exacerbations and are described in detail elsewhere [19, 20]. In brief, both RCTs were conducted in four major paediatric centres in Australia and one in New Zealand. Eligible participants for study enrolment were CYP aged 1–18 years with CT-confirmed bronchiectasis, diagnosed by a respiratory physician, and with at least two respiratory exacerbations in the 18 months before study entry. Exclusion criteria at enrolment, when participants were clinically stable from their disease, included cystic fibrosis, liver dysfunction, beta-lactam or macrolide hypersensitivity, severe (hospitalised) exacerbation within the previous 8 weeks, *Pseudomonas aeruginosa* infection in the previous 4 months, current or prior non-tuberculous mycobacterial infection or previous trial enrolment. Those taking long-term macrolide antibiotics were not excluded, but the antibiotics were discontinued when an exacerbation occurred.

At enrolment, participants had their demographic and medical histories recorded, underwent physical examinations, performed spirometry and a subset provided baseline blood samples, which were stored for subsequent GE analyses. In both RCTs, participants were randomised to the study medications at the onset of their next non-severe exacerbation, which was defined as an increase in cough frequency, change in its character from dry to wet cough or an increase in sputum volume or purulence for three consecutive days. Those with signs of a severe exacerbation (e.g. dyspnoea, hypoxia [SpO2 < 90% in air] or admitted to hospital) were excluded [20].

Participant selection for the current study was based upon sample availability, and prospectively collected clinical data confirming the presence or absence of an exacerbation consistent with the study definition had occurred during the 3-month interval following enrolment and collection of baseline bloods. Twenty-two participants fulfilled these selection criteria and had experienced an exacerbation within the 3-month timeframe, and they were compared with nine who did not have an exacerbation during this period.

Human research ethics committees at each participating site approved the RCTs, and written informed consent was obtained from parents or caregivers, including approval to store biological samples for subsequent analyses.

### Blood collection

Peripheral blood (2.5 mL) was collected into PAXgene Blood RNA tubes (BD Biosciences, USA) from participants at enrolment, when they were in a stable clinical state (baseline sample) and biobanked at −80 °C according to manufacturer’s instructions.

### RNA extraction and transcriptomics analysis

Total RNA was extracted using the corresponding PAXgene Blood RNA Kit (QIAGEN, USA), as per the manufacturer’s protocol, using automated extraction with the QIAcube (Qiagen, Hilden, Germany). Isolated total RNA samples were assayed for quality (Agilent 2100 Bioanalyser, Agilent Technologies, Santa Clara, CA, USA) and quantity (Quant-iT RiboGreen, Life Technologies, Carlsbad, CA, USA). Extracted RNA (500 ng) was reverse transcribed into antisense RNA and biotin-UTP labelled using the Illumina TotalPrep RNA Amplification Kit (Thermofisher Scientific, USA). Purified labelled antisense RNA (750 ng) was then hybridised to the HumanHT-12 v4.0 Expression BeadChip arrays containing > 47,000 probes, as per the manufacturer’s recommended protocols (Illumina, USA). Arrays were scanned using the Illumina BeadArray Reader.

### Transcriptomic statistical analysis

Transcriptomic data were exported using Genome Studio (Illumina, San Diego, USA) and imported and analysed using GeneSpring GX14. Probe-centred data were log-transformed, normalised and baseline converted to the median of all samples. Data were filtered and only genes flagged as present (< 0.05 detection *p* value) were included for further analysis (15,842 entities). Differential GE between exacerbation and non-exacerbation groups was determined using ANOVA, with Tukey post hoc testing (*p* < 0.05 adjusted for multiple comparisons using Benjamini–Hochberg false discovery rate (FDR) correction).

### Real-time quantitative polymerase chain reaction (RT-qPCR) verification

To verify the identified significant DEGs, RT-qPCR was performed utilising the same samples used for the microarray analysis. Lyophilised QuantiTect Primer Assays for genes *ANXA3*, *ALAS2*, *DEFA1*, *ADM*, *ALPL*, *S100A12*, *TNFAIP6*, *SNCA*, *MCEMP1*, *PROK2*, *UBE2DS2* and *HPRT1* were used as per manufacturer’s protocols (QIAGEN, USA). The RT-qPCR reactions were prepared (using 8 ng of total RNA) using QuantiTect SYBR Green RT-PCR Kit (QIAGEN, USA) as per the manufacturer’s Real-Time One-Step RT-PCR protocols with plates analysed on a ViiA 7 real-time thermal cycler (Applied Biosystems, USA). The following cycling conditions were employed: reverse transcription (50 °C for 30-min); PCR initial activation step (95 °C for 15-min); 40 cycles of 94 °C for 15-s (denaturation), 55 °C for 30-s (annealing) and 72 °C for 30-s (extension). The 2^−ΔCT^ method [21] was used to quantify the expression levels of the target genes, with *UBE2DS2* and *HPRT1* used as the housekeeping genes [22, 23].

### Pathway analyses

Functional analysis of the DEGs identified from the microarray results (647 gene entities) was performed using Ingenuity Pathway Analysis (IPA, Ingenuity Systems). A core analysis workflow was selected, which identified generalised pathways, diseases and functions in our dataset. IPA uses Fisher’s exact test at the right tail to calculate a *p* value, with Benjamini–Hochberg correction for multiple testing also applied. For canonical pathway analysis, disease and function, the  −log (*p* value) > 1.3 (*p* value ≤ 0.05) was used as the threshold; the Z-score > 2 was defined as the threshold of significant activation, whilst Z-score <  − 2 was defined as the threshold of significant inhibition [24, 25].

### Statistical analyses

All statistical analyses were performed using GeneSpring GX14 (Agilent Technologies, Santa Clara, USA) and Statistical Package for Social Science (SPSS v27.0; IBM, USA) or Stata (StataCorp v17). Data are presented as counts and percentages, median and interquartile range (IQR) or mean and standard deviation (SD), as appropriate. Group differences in clinical characteristics were analysed by Fisher’s exact test, independent *t*-test or Mann–Whitney *U* test, as appropriate. Spearman’s correlation analysis was performed using continuous data for time-to-next exacerbation with the identified top RT-qPCR differentially expressed genes 2^-ΔCT^ values. Receiver operating characteristic (ROC) curves and area under the curve (AUC) were determined to understand the diagnostic potential these genes have in discriminating between groups with or without an exacerbation.

## Results

### Clinical characteristics of participants

Demographic and clinical data, including examination findings, as well as spirometry results, the white blood cell count and serum C-reactive protein concentrations of the 31 CYP participants were recorded at enrolment (Table 1). Both groups showed similar demographic and clinical baseline medical histories. However, those who had an exacerbation within 3 months of their baseline samples had more bronchiectasis-related hospitalisations in the 2 years prior to enrolment (*p* = 0.04) and a shorter period for time-to-next exacerbations (*p* < 0.001).

**Table 1:** Participant characteristics at enrolment

Characteristics	Exacerbation within 3 months of enrolment (*n* = 22)	No exacerbation within 3 months of enrolment (*n* = 9)	*p* value
*Demographic*			
Age, mean (SD), years	8.9 (4.0)	6.8 (3.9)	0.19
Male sex, *n* (%)	13 (59)	4 (44)	0.69
Indigenous, *n* (%)	1 (4.5)	0	0.52
*Medical history*			
Preterm birth (< 37 weeks’ gestation), *n* (%)	4 (18)	3 (33)	0.64
Birthweight, mean (SD), grams	3365 (1193)	2957 (942)	0.40
Number of lobes affected, median (IQR)	2.5 (2, 4)	3 (2, 4)	0.60
Daily cough, *n* (%)	9 (41)	3 (33)	1.0
Number of non-hospitalised exacerbations in previous 12 months, median (IQR)	3 (1, 5)	2 (1, 5)	0.82
Number of hospitalised exacerbations in previous 2 years, median (IQR)	1 (0.8, 2.3)	0 (0.0, 1.5)	**0.04**
Time-to-next exacerbation (months), median (IQR)	1.5 (0.5, 2.6)	5.5 (4.9, 6.1)	** < 0.001**
Underlying cause of bronchiectasis, *n* (%)			
Idiopathic/post infection Other	16 (73) 6 (27)	8 (89) 1 (11)	0.64
*Anthropometric and spirometry results*			
Weight, median (IQR), kg	26 (16, 33)	36 (20, 56)	0.15
Height, mean (SD), cm	121 (24)	133 (24)	0.20
FEV_1_% predicted, median (IQR)	88 (79, 93)	83 (76, 97)	0.33
FVC% predicted, median (IQR)	88 (79, 98)	88 (79, 99)	0.84
FEV_1_/FVC%, median (IQR)	86 (80, 91)	85 (77, 89)	0.55
FEF_25–75%_, median (IQR)	83 (64, 89)	68 (58, 92)	0.53
*White blood count and CRP results*			
CRP (mg/L), median (IQR)	2 (2, 2)	2 (2, 3.05)	0.12
White blood cell count, ×10^9^/L, median (IQR)	8.2 (7.5, 9.5)	8.6 (7.8, 9.2)	0.85
Neutrophils (×10^9^/L), mean (SD)	3.3 (0.1)	3.6 (0.7)	0.48
Lymphocytes (×10^9^/L), median (IQR)	3.48 (2.85, 4.43)	3.6 (2.72, 3.97)	0.45
Monocytes (×10^9^/L), mean (SD)	0.6 (0.2)	0.7 (0.2)	0.47
Eosinophils (×10^9^/L), mean (SD)	0.5 (0.2)	0.6 (0.4)	0.21
Basophils (×10^9^/L), median (IQR)	0.04 (0.03, 0.05)	0.03 (0.02, 0.04)	0.23

Continuous data were presented as the mean ± standard deviation or median (interquartile range). Categorical data were presented as numbers with percentages. Values in bold indicate *p *< 0.05

*CRP* C-reactive protein, *FEF*_*25–75*_ forced expiratory flow between 25 and 75% of forced vital capacity, *FEV*_*1*_ forced expiratory volume in 1 s, *FVC* forced vital capacity, *IQR* interquartile range, *n* number, *SD* standard deviation

### Differential gene expression and pathways associated with a future exacerbation of bronchiectasis

From the microarray profiling, data were filtered for flags present or marginally present in all samples (*n* = 31), with a total of 15,842 gene entities being identified. We then identified 647 gene entities that were significantly dysregulated (*p* < 0.05) in the exacerbation group (*n* = 22) compared to the non-exacerbation group (*n* = 9, Supplementary Figure 1).

Pathway analysis identified a total of 13 canonical pathways that were predicted to be significantly inhibited, with the most significantly inhibited pathway being neutrophil degranulation (*p* < 0.001) in those who subsequently had an exacerbation within the next 3 months compared to those who did not (Supplementary Table 1).

**Fig. 1:**
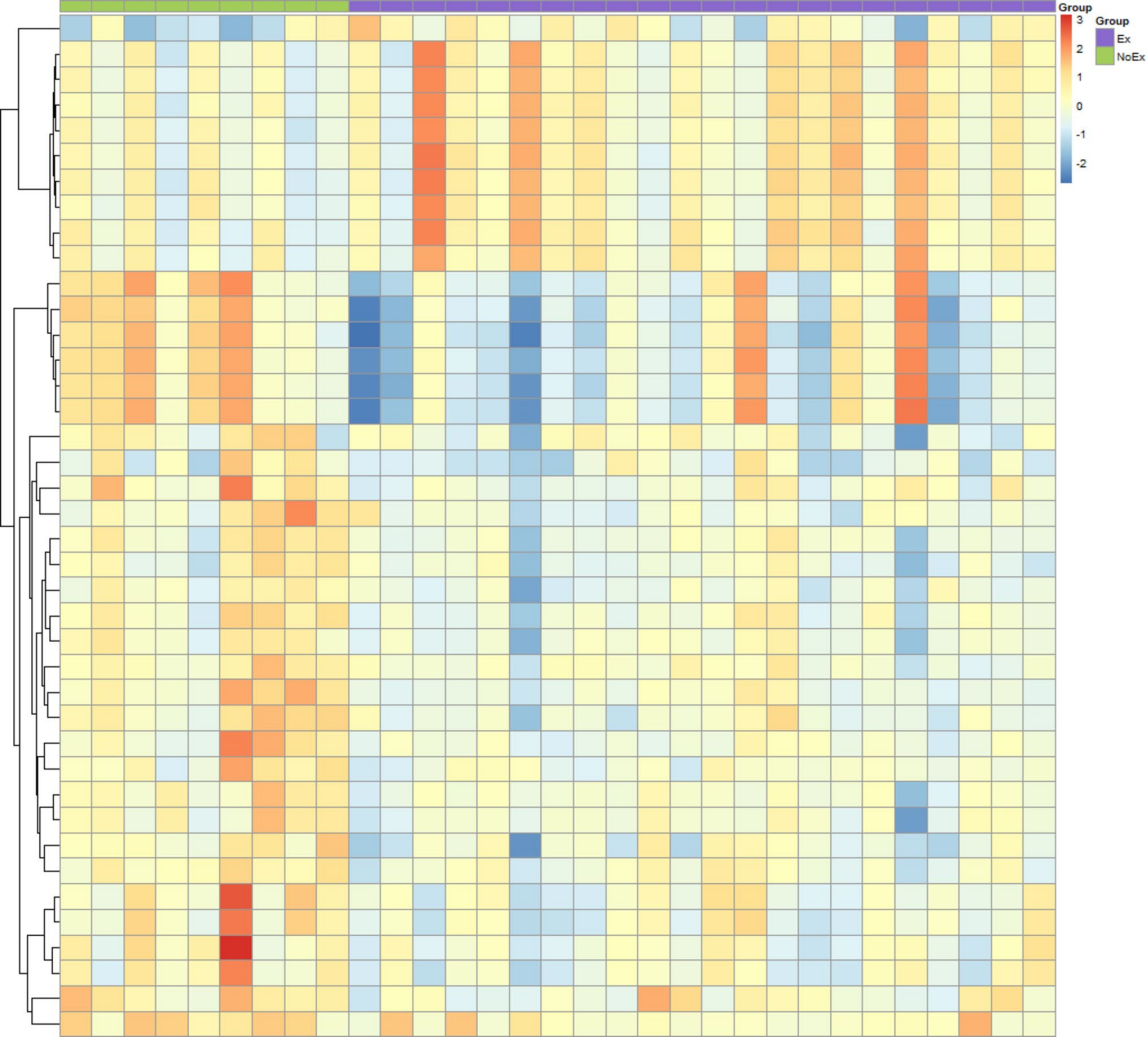
Supervised clustering heatmap of the 40 mRNA entities that were significantly dysregulated (fold change > 1.5, *p* < 0.05) between those who had an exacerbation within 3 months post-baseline sampling at enrolment (*n* = 22, purple) compared to those who did not have an exacerbation during this period (*n* = 9, green). Red intensity indicates normalised over-expression of genes, and blue intensity indicates normalised decreased expression. Ex, exacerbation; NoEx, no exacerbation

To identify the top markers with the most significant differences, the cut-off criteria were further restricted to a 1.5-fold change and significance parameter of *p* ≤ 0.05. The number of significantly dysregulated genes decreased to 40 entities, with 10 entities (9 genes) significantly upregulated and 30 entities (24 genes) significantly downregulated (Fig. 1 and Table 2). The most significantly dysregulated gene was *DEFA1* (downregulated −2.6-fold change, *p* = 0.005). These significant genes overlap with the top targets identified by pathway analysis (Supplementary Table 1).

**Table 2:** Forty significantly expressed mRNA entities identified from microarray assays and corresponding receiver operator curve analysis in baseline blood samples at enrolment from participants who subsequently had an exacerbation within the next 3 months (*n* = 22) compared to participants who did not have an exacerbation during this period (*n* = 9)

Entities	Definition	Regulation	Fold change	*p* value	Area under the curve (95% confidence interval)	*p* value
*ERAP2*	Endoplasmic reticulum aminopeptidase 2 (ERAP2), mRNA	Up	1.72	0.03	0.727 (0.529, 0.925)	0.05
*FAM40B*	Family with sequence similarity 40, member B (FAM40B), mRNA	Up	1.56	0.044	0.712 (0.502, 0.922)	0.068
*C9orf130*	PREDICTED: chromosome 9 open reading frame 130 (C9orf130), mRNA	Up	1.55	0.046	0.727 (0.527, 0.927)	0.05
*DKFZp434K191*	Hypothetical protein DKFZp434K191 (DKFZp434K191), mRNA	Up	1.55	0.044	0.722 (0.533, 0.911)	0.056
*EVI5*	Ecotropic viral integration site 5 (EVI5), mRNA	Up	1.54	0.035	0.747 (0.558, 0.937)	0.033
*PPP2R3A*	Protein phosphatase 2 (formerly 2 A), regulatory subunit B″, alpha (PPP2R3A), transcript variant 2, mRNA	Up	1.54	0.049	0.737 (0.550, 0.925)	0.041
*FAM153B*	Family with sequence similarity 153, member B (FAM153B), mRNA	Up	1.53	0.044	0.717 (0.514, 0.921)	0.061
*ZNF652*	Zinc finger protein 652 (ZNF652), mRNA	Up	1.53	0.033	0.727 (0.522, 0.933)	0.05
	NISC_na09g08.y1 COGENE 8.5 EAT Homo sapiens cDNA clone IMAGE:5794742 5, mRNA sequence	Up	1.51	0.042	0.727 (0.529, 0.926)	0.05
*ALS2CR14*	Amyotrophic lateral sclerosis 2 (juvenile) chromosome region, candidate 14 (ALS2CR14), mRNA	Up	1.51	0.039	0.717 (0.519, 0.915)	0.061
*NAMPT*	Nicotinamide phosphoribosyltransferase (NAMPT), mRNA	Down	−1.51	0.005	0.783 (0.580, 0.985)	0.015
*SCGB3A1*	Secretoglobin, family 3 A, member 1 (SCGB3A1), mRNA	Down	−1.55	0.011	0.753 (0.567, 0.938)	0.03
*SOD2*	Superoxide dismutase 2, mitochondrial (SOD2), nuclear gene encoding mitochondrial protein, transcript variant 2, mRNA	Down	−1.56	0.014	0.742 (0.516, 0.969)	0.037
*CST7*	Cystatin F (leukocystatin) (CST7), mRNA	Down	−1.56	0.007	0.763 (0.540, 0.985)	0.024
*VNN2*	Vanin 2 (VNN2), transcript variant 1, mRNA	Down	−1.57	0.009	0.727 (0.500, 0.954)	0.05
*GCA*	Grancalcin, EF-hand calcium binding protein (GCA), mRNA	Down	−1.57	0.006	0.798 (0.608, 0.988)	0.01
*LOC730235*	PREDICTED: hypothetical LOC730235 (LOC730235), mRNA	Down	−1.57	0.005	0.808 (0.655, 0.962)	0.008
*SOD2*	Superoxide dismutase 2, mitochondrial (SOD2), nuclear gene encoding mitochondrial protein, transcript variant 3, mRNA	Down	−1.59	0.002	0.818 (0.634, 1)	0.006
*NAMPT*	Nicotinamide phosphoribosyltransferase (NAMPT), mRNA	Down	−1.59	0.009	0.813 (0.640,0.987)	0.007
*ACSL1*	Acyl-CoA synthetase long-chain family member 1 (ACSL1), mRNA	Down	−1.59	0.015	0.758 (0.537, 0.978)	0.026
*VNN2*	Vanin 2 (VNN2), transcript variant 1, mRNA	Down	−1.6	0.041	0.783 (0.579, 0.986)	0.015
*ORM1*	Orosomucoid 1 (ORM1), mRNA	Down	−1.6	0.025	0.768 (0.595, 0.940)	0.021
*S100A8*	S100 calcium binding protein A8 (S100A8), mRNA	Down	−1.61	0.033	0.727 (0.527, 0.938)	0.05
*ALAS2*	Aminolevulinate, delta-, synthase 2 (ALAS2), nuclear gene encoding mitochondrial protein, transcript variant 3, mRNA	Down	−1.62	0.026	0.732 (0.540, 0.938)	0.045
*SNCA*	Synuclein, alpha (non A4 component of amyloid precursor) (SNCA), transcript variant NACP112, mRNA	Down	−1.65	0.03	0.712 (0.516, 0.908)	0.068
*PROK2*	Prokineticin 2 (PROK2), mRNA	Down	−1.68	0.006	0.798 (0.601, 0.995)	0.01
*C19orf59*	Chromosome 19 open reading frame 59 (C19orf59), mRNA	Down	−1.71	0.003	0.747 (0.527, 0.968)	0.033
*SNCA*	Synuclein, alpha (non A4 component of amyloid precursor) (SNCA), transcript variant NACP140, mRNA	Down	−1.71	0.016	0.742 (0.560, 0.925)	0.037
*TNFAIP6*	Tumor necrosis factor, alpha-induced protein 6 (TNFAIP6), mRNA	Down	−1.74	0.004	0.773 (0.580, 0.966)	0.019
*ALAS2*	Aminolevulinate, delta-, synthase 2 (ALAS2), nuclear gene encoding mitochondrial protein, transcript variant 2, mRNA	Down	−1.75	0.015	0.747 (0.554, 0.941)	0.033
*S100A12*	S100 calcium binding protein A12 (S100A12), mRNA	Down	−1.77	0.005	0.788 (0.620, 0.956)	0.013
*ALPL*	Alkaline phosphatase, liver/bone/kidney (ALPL), transcript variant 1, mRNA	Down	−1.78	0.009	0.773 (0.603, 0.942)	0.019
*ADM*	Adrenomedullin (ADM), mRNA	Down	−1.89	0.001	0.828 (0.651, 1)	0.005
*ANXA3*	Annexin A3 (ANXA3), mRNA	Down	−1.93	< 0.001	0.869 (0.738, 0.999)	0.001
*LOC653600*	PREDICTED: similar to neutrophil defensin 1 precursor (HNP-1) (HP-1) (HP1) (defensin, alpha 1) (LOC653600), mRNA	Down	−2.35	0.004	0.833 (0.692, 0.975)	0.004
*DEFA1B*	Defensin, alpha 1B (DEFA1B), mRNA	Down	−2.38	0.006	0.808 (0.657, 0.959)	0.008
*DEFA1B*	Defensin, alpha 1B (DEFA1B), mRNA	Down	−2.45	0.008	0.823 (0.677, 0.969)	0.005
*DEFA1B*	Defensin, alpha 1B (DEFA1B), mRNA	Down	−2.48	0.005	0.823 (0.677, 0.970)	0.005
*DEFA3*	Defensin, alpha 3, neutrophil-specific (DEFA3), mRNA	Down	−2.5	0.005	0.828 (0.686, 0.971)	0.005
*DEFA1*	Defensin, alpha 1 (DEFA1), mRNA	Down	−2.6	0.005	0.813 (0.663, 0.963)	0.007

To further evaluate the predictive ability of these 40 significantly dysregulated entities (33 genes total), ROC curve analyses were performed (Table 2). The highest AUC for a single gene was achieved by *ANXA3* (AUC 0.869; 95% confidence interval [CI] 0.738–0.999; *p* = 0.001). All other genes had an AUC that ranged from 0.712 to 0.833.

### Verification of microarray data by RT-qPCR analysis

**Fig. 2:**
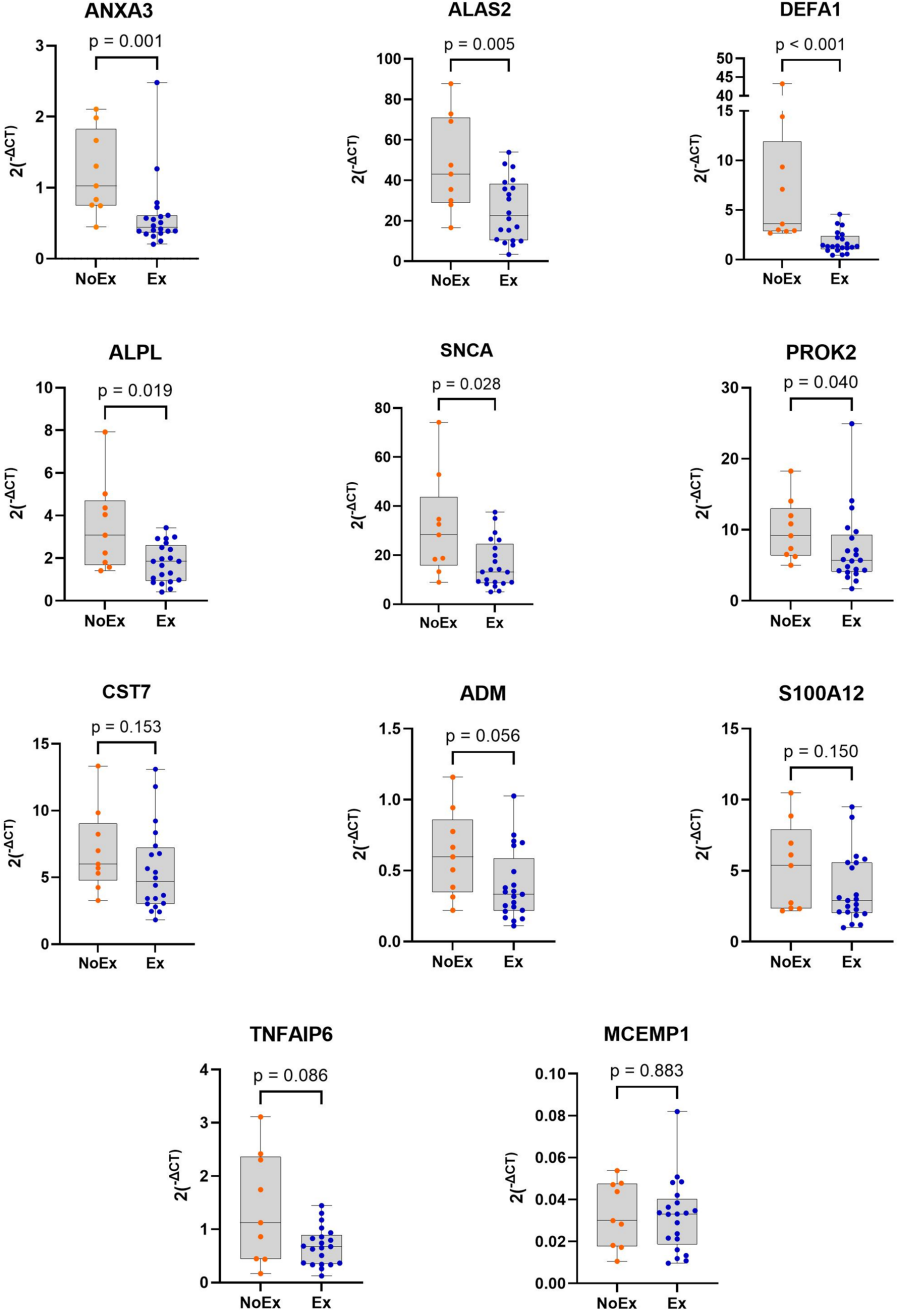
RT-qPCR verification of the top 11 significantly dysregulated genes from pathway and network analyses in those who had an exacerbation (blue) compared to those who did not (orange) within 3 months of their baseline sampling at enrolment. Ex, exacerbation; NoEx, no exacerbation; RT-qPCR, real-time quantitative polymerase chain reaction

To verify the microarray results, we performed a RT-qPCR utilising the same samples as used for the microarray assays for 11 genes (*ANXA3*,* ALAS2*, *CST7*, *DEFA1*, *ADM*, *ALPL*, *S100A12*, *TNFAIP6*, *SNCA*, *MCEMP1*, *PROK2*) that showed the highest DEG and were also identified as top genes from the pathway network analyses. Whilst RT-qPCR verification showed the same direction of dysregulation (under-expressed) as the microarray analysis, only six genes (*ANXA3*, *ALAS2*, *DEFA1*, *ALPL*, *SNCA* and *PROK2*) were significantly different between the groups (Fig. 2). Correlation analysis was also performed against the top 11 genes (Table 3). Six of these 11 genes were significantly positively correlated with a shorter time-to-next exacerbation (*ANXA3*, *ALAS2*, *DEFA1*, *ALPL*, *PROK2*, *CST7*).

**Table 3:** Spearman’s rank correlation analysis for the 11 genes identified as the top genes from the pathway network analyses and used for RT-qPCR verification

Gene	Spearman’s rho	*p* value
*ANXA3*	0.556	0.002
*ALAS2*	0.446	0.015
*DEFA1*	0.471	0.009
*ALPL*	0.430	0.018
*SNCA*	0.360	0.051
*PROK2*	0.503	0.005
*CST7*	0.515	0.004
*ADM*	0.344	0.062
*S100A12*	0.293	0.115
*TNFAIP6*	0.244	0.194
*MCEMP1*	0.194	0.437

### Discriminatory assessment of six internally validated genes

Finally, we assessed the AUC (Table 4) for the six genes that were successful in the verification by RT-qPCR (*ANXA3*, *ALAS2*, *DEFA1*, *ALPL*, *SNCA*, *PROK2*). The AUC ranged from 0.728 to 0.917, with DEFA1 as the best performing gene (AUC 0.92, 95% CI 0.82, 1.00).

**Table 4:** AUC analysis for the six genes successfully verified by RT-qPCR as significantly associated with exacerbation within the next 3 months. Comparative microarray AUC results are also included

Gene	Original microarray AUC (95%CI)	*p* value	RT-qPCR AUC (95%CI)	*p* value
*ANXA3*	0.87 (0.738, 0.999)	0.001	0.87 (0.73, 1.00)	0.002
*ALAS2*	0.75 (0.554, 0.941)	0.033	0.77 (0.59, 0.95)	0.024
*DEFA1*	0.81 (0.663, 0.963)	0.007	0.92 (0.82, 1.00)	0.0003
*ALPL*	0.77 (0.603, 0.942)	0.019	0.76 (0.56, 0.96)	0.027
*SNCA*	0.74 (0.560, 0.925)	0.037	0.74 (0.55, 0.94)	0.038
*PROK2*	0.80 (0.601, 0.995)	0.01	0.73 (0.55, 0.91)	0.053

*AUC* area under the curve, *RT-qPCR* real-time quantitative polymerase chain reaction

## Discussion

Our study identified a peripheral blood GE profile associated with an exacerbation within the next 3 months in CYP with bronchiectasis. Microarray and pathway analysis determined neutrophil degranulation was the dominant affected pathway, which was significantly inhibited, and subsequent RT-qPCR verification found six genes, *ANXA3*, *ALAS2*, *DEFA1*, *ALPL*, *SNCA*, *PROK2*, were discriminatory at identifying children at risk of an imminent exacerbation. Five of these genes were further positively correlated with a shorter time-to-next exacerbation (*ANXA3*, *ALAS2*, *DEFA1*, *ALPL*, *PROK2*).

Bronchiectasis exacerbations place a high burden on the patient, their families [26] and healthcare system [11]. According to an international survey, they are also among the top clinical and research priorities reported by patients, parents of children with bronchiectasis and clinicians [27]. Hence, it is unsurprising that reducing exacerbations is one of the key goals of bronchiectasis management [1], especially as a lower exacerbation rate in children is associated with a greater likelihood of radiographic resolution of bronchiectasis [28]. Whilst GE signatures associated with exacerbation risk are reported in chronic adult airway diseases [17, 29], including in sputum samples from adults with bronchiectasis [30], these have not been investigated previously in paediatric bronchiectasis.

Persistent neutrophilic inflammation is a key feature of bronchiectasis and is the dominant inflammatory endotype in adult [14] and paediatric [15] bronchiectasis. It was therefore not surprising that the top pathway and key identified genes found in this study were neutrophil-associated. Specifically, the biological pathway ‘neutrophil degranulation’ was significantly dysregulated in those experiencing an exacerbation within the following 3 months. Furthermore, this impairment of neutrophil degranulation is also described in adult bronchiectasis where it is associated with more severe disease [31, 32]. Reprogrammed peripheral blood neutrophils showing prolonged survival due to impaired apoptosis, increasing and often dysfunctional NETosis, as well as decreased functional ability to phagocytose bacteria have been found in stable adult bronchiectasis patients [33]. In turn, this perpetuates the vicious vortex often described in bronchiectasis [34]. Indeed, our study supports the concept of neutrophil reprogramming also occurring in paediatric bronchiectasis, something which will need further exploration.

The top neutrophil-associated gene product, Annexin A3 (ANXA3), is a calcium-dependent phospholipid and membrane-binding protein associated with cytoplasmic granules in neutrophils and monocytes [35]. We identified under-expression of *ANXA3* in those having an exacerbation within 3 months following their baseline enrolment blood sample. ANXA3 may play a role in paediatric bronchiectasis similar to that described in sepsis [36], where it has a beneficial role during the early phase of the immune response by increasing pathogen clearance but then has a later detrimental role (neutrophils become ‘reprogrammed’) by prolonging neutrophil survival and further contributing to tissue injury. ANXA3 plays a critical role in regulating apoptotic events [36]. When ANXA3 is reduced, the balance of apoptosis-related proteins shifts, potentially leading to impaired apoptotic cell clearance [37]. As frequent exacerbations are associated with chronic airway inflammation, often damaging epithelial junctions and causing structural remodelling, reduced *ANXA3* expression may lead to an exhausted state where the protective, restorative functions of ANXA3 are therefore reduced or lost.

*DEFA1* encodes one of the subtypes of α-defensins, also referred to as human neutrophil peptides, which are major constituents of neutrophil azurophilic granules where they play a crucial role in antimicrobial activity and inflammation in the lungs [38]. We have shown previously that α-defensins are upregulated in children with protracted bacterial bronchitis [39], a chronic lower airway disorder at the start of the spectrum that can lead to bronchiectasis [1]. Chronic neutrophilic inflammation, as seen in adult bronchiectasis, is perpetuated by a reprogrammed phenotype whereby neutrophil apoptosis is delayed [33]. Our results suggest that *DEFA1* plays a key role in the pathophysiology of paediatric bronchiectasis, where even during the stable clinical state, the gene dysregulation contributes to the dysfunction and prolonged survival of neutrophils, resulting in failure to resolve inflammation, further leading to persistent infection and structural lung damage.

Alkaline phosphatases are plasma membrane-bound glycoproteins and the *ALPL* gene encodes neutrophil alkaline phosphatase (ALPL), which increases during bacterial infections and is associated with enhanced neutrophil chemotaxis, inflammation, reactive oxygen species production and apoptosis [40]. ALPL’s role in the TNF-α/nuclear factor κB pathway has also been suggested to contribute to neutrophilic inflammation in asthmatic patients [29].

Protein 5′-aminolevulinate synthase 2 (ALAS2) is an erythrocyte-specific mitochondrial localisation enzyme that regulates the initial step of haem biosynthesis [41], which has been associated with a number of respiratory diseases such as cystic fibrosis, COPD and acute respiratory distress syndrome [42]. Additionally, free haem is an inflammatory molecule and has been shown to induce the activation and migration of neutrophils [43]. Alpha-(α)-synuclein, encoded by *SNCA*, has been positively correlated with neutrophils, macrophages and dendritic cells, as well as immune cell marker genes (*FPR1* and *SIGLEC5* in neutrophils) [44]. The final gene, Prokineticin 2 (*PROK2*), regulates bone marrow and peripheral blood [45] and is a marker of a lung neutrophil subset in acute respiratory distress syndrome [46].

All six genes are related to neutrophil function, yet all six genes were downregulated in those experiencing an exacerbation within 3 months following their baseline sample at enrolment compared to those who did not have an exacerbation during the same period. It is unclear why these children had a reduced neutrophil GE signature at their baseline. A study investigating mechanisms underlying adaptive changes in cystic fibrosis airway neutrophils also reported pathways that were significantly downregulated included genes belonging to the neutrophil degranulation pathway, suggesting a relationship between transcription of these genes and impaired neutrophil ability to clear pathogens [47]. Therefore, reduced GE may translate to reduced neutrophil-mediated immunity and vulnerability to an exacerbation (i.e. bacterial clearance). Given the group who went on to experience an exacerbation were also more likely to have had severe exacerbations requiring hospitalisations in the previous 2 years (Table 1), we speculate that this group had a ‘reprogramming of neutrophils’ [33] contributing to aberrant inflammation with reduced microbial killing. Future research is required to understand the role of these genes in bronchiectasis exacerbations and whether they represent a particular disease endotype.

Our study has several strengths. We consider a key aspect is identifying six neutrophil-related genes including *DEFA1*, *ANXA3*, *ALAS2*, *ALPL*, *SNCA* and *PROK2* associated with CYP who subsequently underwent an exacerbation compared to those who did not have an exacerbation within the same 3-month period. To our knowledge, this is the first study to identify a neutrophilic GE profile in a clinically stable paediatric bronchiectasis cohort that has the potential to discriminate those at high risk of an exacerbation within the next few months. Furthermore, these significantly dysregulated genes were identified from blood samples, which is of clinical importance in a paediatric setting as they are minimally invasive diagnostic tests and present less risk and discomfort to patients than invasive sampling, such as bronchoalveolar lavage or even sputum induction.

Nevertheless, our study has several limitations. Although we had detailed clinical information and identified significantly dysregulated genes using microarray technology and internally verified them in the same cohort using RT-qPCR technology, our cohort was small, raising the possibility of type I errors. We also lacked an independent validation cohort. Furthermore, validation of protein levels for the identified top DEGs with ELISAs or other protein quantification methods to support these markers is required. All of which means this is a ‘proof-of-concept study’ and a much larger and independent patient cohort is needed to validate the expression of these six hub genes. Whilst microarray technology allowed us to investigate GE in paediatric bronchiectasis and generate a list of DEGs, further pathway analyses are required. Despite having performed basic analyses, we acknowledge more in-depth pathway analysis is required on a larger scale. Finally, future experiments utilising RNA-sequencing technology may allow superior detection of low abundance transcripts, differentiating biologically critical isoforms and identifying genetic variants, as well as avoiding technical issues that can arise in microarray studies due to probe performance [48].

In conclusion, we have provisionally identified that peripheral blood holds specific biological information which can potentially identify an imminent risk of future exacerbations in children with bronchiectasis. The six significantly dysregulated genes are associated with neutrophils, and future studies will need to examine whether they are involved in the pathogenesis of exacerbations or represent markers of those likely to exacerbate. Validating these peripheral blood neutrophil-associated genes has the potential to support clinical care by enabling a precision medicine approach to earlier targeted treatment.

## Supplementary Information

Below is the link to the electronic supplementary material.

Supplementary file1 (DOCX 17.2 KB)

Supplementary file2 (DOCX 56.9 KB)

## Data Availability

The data that support the findings of this study are available on request from the corresponding author. The data are not publicly available due to privacy or ethical restrictions.

## References

[1] Chang AB, Bush A, Grimwood K (2018) Bronchiectasis in children: diagnosis and treatment. Lancet 392(10150):866–879 30215382 10.1016/S0140-6736(18)31554-X

[2] Chang AB, Bell SC, Byrnes CA, Dawkins P, Holland AE, Kennedy E et al (2023) Thoracic Society of Australia and New Zealand (TSANZ) position statement on chronic suppurative lung disease and bronchiectasis in children, adolescents and adults in Australia and New Zealand. Respirology 28(4):339–349 36863703 10.1111/resp.14479 PMC10947421

[3] Martinez-Garcia MA, Polverino E, Aksamit T (2018) Bronchiectasis and chronic airway disease: it is not just about asthma and COPD. Chest 154(4):737–739 30290922 10.1016/j.chest.2018.02.024

[4] Blackall SR, Hong JB, King P, Wong C, Einsiedel L, Rémond MGW et al (2018) Bronchiectasis in indigenous and non-indigenous residents of Australia and New Zealand. Respirology 23(8):743–749 29502335 10.1111/resp.13280

[5] Roberts JM, Goyal V, Kularatna S, Chang AB, Kapur N, Chalmers JD et al (2023) The economic burden of bronchiectasis: a systematic review. Chest 164(6):1396–1421 37423293 10.1016/j.chest.2023.06.040

[6] Roberts JM, Newcombe PA, Goyal V, Kularatna S, McPhail SM, Chang AB, et al. Development and validation of a parent proxy bronchiectasis child quality of life instrument: The BC-QoL. ERJ Open Res. 2026:01443–2025. 10.1183/23120541.01443-2025 PMC13358714 42445236

[7] Chalmers JD, Chang AB, Chotirmall SH, Dhar R, McShane PJ (2018) Bronchiectasis. Nat Rev Dis Primers 4(1):45 30442957 10.1038/s41572-018-0042-3

[8] Chang AB, Dharmage SC, Marchant JM, McCallum GB, Morris PS, Schultz A et al (2024) Improving the diagnosis and treatment of paediatric bronchiectasis through research and translation. Arch Bronconeumol 60(6):364–373 38548577 10.1016/j.arbres.2024.03.003

[9] Amati F, Simonetta E, Gramegna A, Tarsia P, Contarini M, Blasi F et al (2019) The biology of pulmonary exacerbations in bronchiectasis. European respiratory review : an official journal of the European Respiratory Society 28(154):190055 31748420 10.1183/16000617.0055-2019 PMC9488527

[10] Kapur N, Masters IB, Chang AB (2010) Longitudinal growth and lung function in pediatric non-cystic fibrosis bronchiectasis: what influences lung function stability? Chest 138(1):158–164 20173055 10.1378/chest.09-2932

[11] Goyal V, McPhail SM, Hurley F, Grimwood K, Marchant JM, Masters IB et al (2020) Cost of hospitalization for bronchiectasis exacerbation in children. Respirology 25(12):1250–1256 32358912 10.1111/resp.13828

[12] Chang AB, Boyd J, Bell L, Goyal V, Masters IB, Powell Z et al (2021) Clinical and research priorities for children and young people with bronchiectasis: an international roadmap. ERJ Open Res 7(3):00122-2021 34291113 10.1183/23120541.00122-2021 PMC8287136

[13] De Angelis A, Johnson ED, Sutharsan S, Aliberti S (2024) Exacerbations of bronchiectasis. Eur Respir Rev 33(173):240085 39048130 10.1183/16000617.0085-2024 PMC11267293

[14] Giam YH, Shoemark A, Chalmers JD (2021) Neutrophil dysfunction in bronchiectasis: an emerging role for immunometabolism. Eur Respir J 58(2):2003157 33509959 10.1183/13993003.03157-2020

[15] de Vries JJV, Chang AB, Marchant JM (2018) Comparison of bronchoscopy and bronchoalveolar lavage findings in three types of suppurative lung disease. Pediatr Pulmonol 53(4):467–474 29405664 10.1002/ppul.23952

[16] Shoemark A, Cant E, Carreto L, Smith A, Oriano M, Keir HR et al (2019) A point-of-care neutrophil elastase activity assay identifies bronchiectasis severity, airway infection and risk of exacerbation. Eur Respir J 53(6):1900303 31151955 10.1183/13993003.00303-2019

[17] Baines KJ, Fu JJ, McDonald VM, Gibson PG (2017) Airway gene expression of IL-1 pathway mediators predicts exacerbation risk in obstructive airway disease. Int J Chron Obstruct Pulmon Dis 12:541–550 28223794 10.2147/COPD.S119443 PMC5308595

[18] Fricker M, Gibson PG, Powell H, Simpson JL, Yang IA, Upham JW et al (2019) A sputum 6-gene signature predicts future exacerbations of poorly controlled asthma. J Allergy Clin Immunol 144(1):51-60.e11 30682452 10.1016/j.jaci.2018.12.1020

[19] Goyal V, Grimwood K, Ware RS, Byrnes CA, Morris PS, Masters IB et al (2019) Efficacy of oral amoxicillin-clavulanate or azithromycin for non-severe respiratory exacerbations in children with bronchiectasis (BEST-1): a multicentre, three-arm, double-blind, randomised placebo-controlled trial. Lancet Respir Med 7(9):791–801 31427252 10.1016/S2213-2600(19)30254-1 PMC7172658

[20] Goyal V, Grimwood K, Byrnes CA, Morris PS, Masters IB, Ware RS et al (2018) Amoxicillin-clavulanate versus azithromycin for respiratory exacerbations in children with bronchiectasis (BEST-2): a multicentre, double-blind, non-inferiority, randomised controlled trial. Lancet (London, England) 392(10154):1197–1206 30241722 10.1016/S0140-6736(18)31723-9 PMC7159066

[21] Schmittgen TD, Livak KJ (2008) Analyzing real-time PCR data by the comparative CT method. Nat Protoc 3(6):1101–1108 18546601 10.1038/nprot.2008.73

[22] Roy JG, McElhaney JE, Verschoor CP (2020) Reliable reference genes for the quantification of mRNA in human T-cells and PBMCs stimulated with live influenza virus. BMC Immunol 21(1):4 32005148 10.1186/s12865-020-0334-8 PMC6995044

[23] Oturai DB, Sondergaard HB, Bornsen L, Sellebjerg F, Christensen JR (2016) Identification of suitable reference genes for peripheral blood mononuclear cell subset studies in multiple sclerosis. Scand J Immunol 83(1):72–80 26395032 10.1111/sji.12391

[24] Shao Z, Wang K, Zhang S, Yuan J, Liao X, Wu C et al (2020) Ingenuity pathway analysis of differentially expressed genes involved in signaling pathways and molecular networks in RhoE gene-edited cardiomyocytes. Int J Mol Med 46(3):1225–1238 32705255 10.3892/ijmm.2020.4661 PMC7388835

[25] Krämer A, Green J, Pollard J Jr, Tugendreich S (2013) Causal analysis approaches in ingenuity pathway analysis. Bioinformatics 30(4):523–530 24336805 10.1093/bioinformatics/btt703 PMC3928520

[26] Kapur N, Masters IB, Newcombe P, Chang AB (2012) The burden of disease in pediatric non-cystic fibrosis bronchiectasis. Chest 141(4):1018–1024 21885727 10.1378/chest.11-0679

[27] Chang AB, Boyd J, Bell L, Goyal V, Masters IB, Powell Z et al (2021) Clinical and research priorities for children and young people with bronchiectasis: an international roadmap. ERJ Open Res 7(3):00122–02021 34291113 10.1183/23120541.00122-2021 PMC8287136

[28] Mills DR, Masters IB, Yerkovich ST, McEniery J, Kapur N, Chang AB et al (2024) Radiographic outcomes in pediatric bronchiectasis and factors associated with reversibility. Am J Respir Crit Care Med 210(1):97–107 38631023 10.1164/rccm.202402-0411OC

[29] Baines KJ, Simpson JL, Bowden NA, Scott RJ, Gibson PG (2010) Differential gene expression and cytokine production from neutrophils in asthma phenotypes. Eur Respir J 35(3):522–531 19797135 10.1183/09031936.00027409

[30] Gao Y, Richardson H, Dicker AJ, Barton A, Kuzmanova E, Shteinberg M et al (2024) Endotypes of exacerbation in bronchiectasis: an observational cohort study. Am J Respir Crit Care Med 210(1):77–86 38717347 10.1164/rccm.202310-1729OC

[31] Chalmers JD, Ma A, Turnbull K, Doherty C, Govan JRW, Hill AT (2013) Impaired neutrophil phagocytosis and receptor expression in non-CF bronchiectasis. Eur Respir J 42:2065

[32] Voglis S, Quinn K, Tullis E, Liu M, Henriques M, Zubrinich C et al (2009) Human neutrophil peptides and phagocytic deficiency in bronchiectatic lungs. Am J Respir Crit Care Med 180(2):159–166 19406984 10.1164/rccm.200808-1250OC PMC2714819

[33] Bedi P, Davidson DJ, McHugh BJ, Rossi AG, Hill AT (2018) Blood neutrophils are reprogrammed in bronchiectasis. Am J Respir Crit Care Med 198(7):880–890 29733693 10.1164/rccm.201712-2423OC PMC6173062

[34] Flume PA, Chalmers JD, Olivier KN (2018) Advances in bronchiectasis: endotyping, genetics, microbiome, and disease heterogeneity. Lancet (London, England) 392(10150):880–890 30215383 10.1016/S0140-6736(18)31767-7 PMC6173801

[35] Le Cabec V, Maridonneau-Parini I. Annexin 3 is associated with cytoplasmic granules in neutrophils and monocytes and translocates to the plasma membrane in activated cells. Biochem J. 1994;303 ( Pt 2)(Pt 2):481–7. 10.1042/bj3030481 PMC1137353 7526843

[36] Toufiq M, Roelands J, Alfaki M, Syed Ahamed Kabeer B, Saadaoui M, Lakshmanan AP et al (2020) Annexin A3 in sepsis: novel perspectives from an exploration of public transcriptome data. Immunology 161(4):291–302 32682335 10.1111/imm.13239 PMC7692248

[37] Vandivier RW, Fadok VA, Hoffmann PR, Bratton DL, Penvari C, Brown KK et al (2002) Elastase-mediated phosphatidylserine receptor cleavage impairs apoptotic cell clearance in cystic fibrosis and bronchiectasis. J Clin Invest 109(5):661–670 11877474 10.1172/JCI13572 PMC150889

[38] Zhang H, Porro G, Orzech N, Mullen B, Liu M, Slutsky AS (2001) Neutrophil defensins mediate acute inflammatory response and lung dysfunction in dose-related fashion. Am J Physiol-Lung Cell Mol Physiol 280(5):L947–L954 11290519 10.1152/ajplung.2001.280.5.L947

[39] Baines KJ, Upham JW, Yerkovich ST, Chang AB, Marchant JM, Carroll M et al (2014) Mediators of neutrophil function in children with protracted bacterial bronchitis. Chest 146(4):1013–1020 24874501 10.1378/chest.14-0131

[40] Li H, Zhao Y, Li W, Yang J, Wu H (2016) Critical role of neutrophil alkaline phosphatase in the antimicrobial function of neutrophils. Life Sci 157:152–157 27287680 10.1016/j.lfs.2016.06.005

[41] Ajioka RS, Phillips JD, Kushner JP (2006) Biosynthesis of heme in mammals. Biochim Biophys Acta 1763(7):723–736 16839620 10.1016/j.bbamcr.2006.05.005

[42] Neves J, Haider T, Gassmann M, Muckenthaler MU (2019) Iron homeostasis in the lungs-a balance between health and disease. Pharmaceuticals (Basel) 12(1):5 30609678 10.3390/ph12010005 PMC6469191

[43] Quintela-Carvalho G, Luz NF, Celes FS, Zanette DL, Andrade D, Menezes D et al (2017) Heme drives oxidative stress-associated cell death in human neutrophils infected with Leishmania infantum. Front Immunol 8:1620 29218050 10.3389/fimmu.2017.01620 PMC5703736

[44] Zhang X, Wu Z, Ma K (2022) SNCA correlates with immune infiltration and serves as a prognostic biomarker in lung adenocarcinoma. BMC Cancer 22(1):406 35421944 10.1186/s12885-022-09289-7 PMC9009002

[45] Negri L, Ferrara N (2018) The prokineticins: neuromodulators and mediators of inflammation and myeloid cell-dependent angiogenesis. Physiol Rev 98(2):1055–1082 29537336 10.1152/physrev.00012.2017

[46] Wang K, Wang M, Liao X, Gao S, Hua J, Wu X et al (2022) Locally organised and activated Fth1(hi) neutrophils aggravate inflammation of acute lung injury in an IL-10-dependent manner. Nat Commun 13(1):7703 36513690 10.1038/s41467-022-35492-y PMC9745290

[47] Margaroli C, Moncada-Giraldo D, Gulick DA, Dobosh B, Giacalone VD, Forrest OA et al (2021) Transcriptional firing represses bactericidal activity in cystic fibrosis airway neutrophils. Cell Rep Med 2(4):100239 33948572 10.1016/j.xcrm.2021.100239 PMC8080108

[48] Zhao S, Fung-Leung WP, Bittner A, Ngo K, Liu X (2014) Comparison of RNA-seq and microarray in transcriptome profiling of activated T cells. PLoS One 9(1):e78644 24454679 10.1371/journal.pone.0078644 PMC3894192

