# Supplementary material for: Neutrophil-related gene expression profile is associated with future paediatric bronchiectasis exacerbations
Source: J Mol Med (Berl). 2026 Apr 1;104(1):59. doi: 10.1007/s00109-026-02662-0 (PMC13038636; doi:10.1007/s00109-026-02662-0)
Supplement: Supplementary file 1 — Supplementary file1 (DOCX 17.2 KB) [file 109_2026_2662_MOESM1_ESM.docx]

**Supplementary Table 1.** Top 14 canonical pathways (log(p-value) ≥ 1.3 and a Z-score
>-2 or 2) from the 647 gene entities that were identified to be significantly dysregulated in the participants who had an exacerbation within 3-months of their baseline samples at enrolment.

| **Ingenuity Canonical Pathways** | **-log**  **(p-value)** | **Ratio** | **z-score** | **Molecules** |
| --- | --- | --- | --- | --- |
| Neutrophil degranulation | 13.5 | 0.092 | -4.52 | *ARG1,ASAH1,AZU1,BRI3,BST1,CAMP,CKAP4,CR1,CREG1,CTSB,CYSTM1,DEFA1,DEFA4,DOK3,ELANE,FCER1G,FCGR2A,GCA,GPR84,HPSE,HSPA6,LCN2,LGALS3,LYZ,MAPK14,MCEMP1,MGAM,MGST1,ORM1,P2RX1,PDXK,PGLYRP1,PLAUR,RAB37,RHOF,S100A11,S100A12,S100A8,SLC2A3,STOM,TMC6,TNFAIP6,TUBB,VAPA* |
| NOD1/2 Signalling Pathway | 4.48 | 0.084 | -3.32 | *BIRC3,DEFA1,DEFA1B,DEFA3,DEFA4,HSPA6,IL1B,MAP2K3,MAPK14,OSM,TLR1,TLR5,TLR6,TNFSF13B,TNFSF14* |
| Pyroptosis Signalling Pathway | 4.18 | 0.111 | -2.33 | *AIM2,CASP4,DHX9,IL1B,MAPK14,MEFV,NLRC4,TLR1,TLR5,TLR6* |
| Toll-like Receptor Signalling | 3.99 | 0.117 | -2.45 | *IL1B,IL1RN,IRAK3,MAP2K3,MAPK14,TLR1,TLR5,TLR6,UBB* |
| Antimicrobial peptides | 2.94 | 0.122 | -2.45 | *CAMP,ELANE,LCN2,LYZ,PGLYRP1,S100A8* |
| Pathogen Induced Cytokine Storm Signaling Pathway | 2.76 | 0.053 | -4.00 | *AIM2,CLEC7A,CSF2RA,CSF2RB,IL1B,IL1RN,MAPK14,NLRC4,OSM,SLC2A14,SLC2A3,SOCS3,SRGN,TLR1,TLR5,TLR6,TNFSF13B,TNFSF14* |
| MyD88:MAL(TIRAP) cascade initiated on plasma membrane | 2.47 | 0.119 | -2.24 | *IRAK3,S100A8,TLR1,TLR6,UBB* |
| Role of Pattern Recognition Receptors in Recognition of Bacteria and Viruses | 2.06 | 0.063 | -2.24 | *CLEC7A,IL1B,NLRC4,OSM,TLR1,TLR5,TLR6,TNFSF13B,TNFSF14* |
| Role of Macrophages, Fibroblasts and Endothelial Cells in Rheumatoid Arthritis | 2.05 | 0.049 | -2.84 | *CREB5,F2RL1,FCGR1A,IL1B,IL1RN,IRAK3,MAP2K3,MAPK14,NFATC3,OSM,SOCS3,TLR1,TLR5,TLR6,TNFSF13B* |
| Parkinson's Signalling Pathway | 1.91 | 0.048 | -3.05 | *FCGR1A,FCGR2A,FCGR2C,IL1B,MAPK14,OSM,SNCA,TLR1,TLR5,TLR6,TNFSF13B,TNFSF14,TUBB,UBB* |
| Communication between Innate and Adaptive Immune Cells | 1.89 | 0.068 | -2.45 | *FCER1G,IL1B,IL1RN,TLR1,TLR5,TLR6,TNFSF13B* |
| Activin Inhibin Signalling Pathway | 1.73 | 0.052 | -3.16 | *IL1B,IL1RN,LIMK2,MAF,MAP2K3,MAPK14,TLR1,TLR5,TLR6,TNFSF13B* |
| TREM1 Signalling | 1.56 | 0.071 | -2.24 | *IL1B,NLRC4,TLR1,TLR5,TLR6* |
| Systemic Lupus Erythematosus in T Cell Signalling Pathway | 1.33 | 0.049 | 2.33 | *CAMK4,CASP4,CREB5,FCER1G,GNB1L,MAP2K3,PPP2R3A,RHOF,RHOT2,RPTOR* |
