# Supplementary material for: Neutrophil-related gene expression profile is associated with future paediatric bronchiectasis exacerbations
Source: J Mol Med (Berl). 2026 Apr 1;104(1):59. doi: 10.1007/s00109-026-02662-0 (PMC13038636; doi:10.1007/s00109-026-02662-0)
Supplement: Supplementary file 2 — Supplementary file2 (DOCX 56.9 KB) [file 109_2026_2662_MOESM2_ESM.docx]

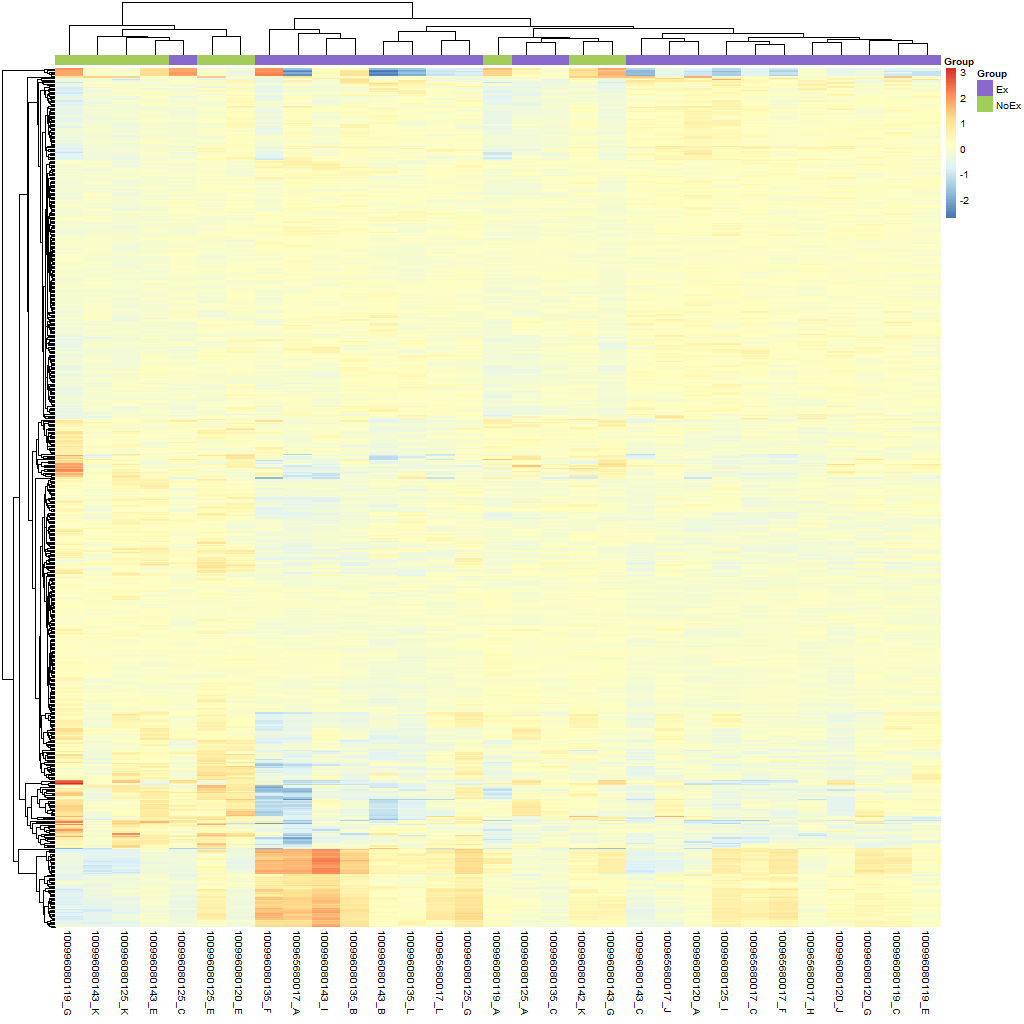


**Supplementary Figure 1.** Unsupervised clustering heatmap of the 647 entities that were significantly dysregulated (p<0.05) between those who did (n=22, purple) and those who did not have an exacerbation within 3-months of enrolment (n=9, green). Red intensity indicates normalised over-expression of genes, and blue intensity indicates normalised decreased expression. Ex, exacerbation; NoEx, no exacerbation.
